# Influence of Microplastics on the Growth and the Intestinal Microbiota Composition of Brine Shrimp

**DOI:** 10.3389/fmicb.2021.717272

**Published:** 2021-09-29

**Authors:** Hongyu Li, Hongwei Chen, Jiao Wang, Jiayao Li, Sitong Liu, Jianbo Tu, Yanzhen Chen, Yanping Zong, Pingping Zhang, Zhiyun Wang, Xianhua Liu

**Affiliations:** ^1^School of Environmental Science and Engineering, Tianjin University, Tianjin, China; ^2^Tianjin Marine Environment Monitoring Center Station of State Oceanic Administration, Tianjin, China; ^3^College of Food Science and Engineering, Tianjin Agricultural University, Tianjin, China

**Keywords:** microplastics, polyethylene, polystyrene, Artemia, biotoxicity, intestinal microbes

## Abstract

Microplastics (MPs) are ubiquitous in the aquatic environment and can be frequently ingested by zooplankton, leading to various effects. Brine shrimp (*Artemia parthenogenetica*) has an important role in the energy flow through trophic levels in different seawater systems. In this work, the influence of polyethylene (PE) and polystyrene (PS) MPs on the growth of brine shrimp and corresponding changes of gut microbiota were investigated. Our results showed that the MPs remarkably reduced the growth rate of brine shrimp, and the two types of MPs have different impacts. The average body length of brine shrimps was reduced by 17.92 and 14.95% in the PE group and PS group, respectively. MPs are mainly found in the intestine, and their exposure evidently affects the gut microbiota. By using 16S rRNA gene high-throughput sequencing, 32 phyla of bacteria were detected in the intestine, and the microbiome consisted mainly of Proteobacteria, Firmicutes, and Actinobacteria. MPs’ exposure significantly increased the gut microbial diversity. For the PE group, the proportion of Actinobacteria and Bacteroidetes increased by 45.26 and 2.73%, respectively. For the PS group, it was 54.95 and 1.27%, respectively. According to the analysis on genus level, the proportions of *Ponticoccus*, *Seohaeicola*, *Polycyclovorans*, and *Methylophaga* decreased by 46.38, 1.24, 1.07, and 2.66%, respectively, for the PE group and 57.87, 1.43, 0.88, and 2.24%, respectively, for the PS group. In contrast, the proportions of *Stappia*, *Microbacterium*, and *Dietzia* increased by 1.12, 23.27, and 11.59%, respectively, for the PE group, and 1.09, 3.79, and 42.96%, respectively, for the PS group. These experimental results demonstrated that the ingestion of MPs by brine shrimp can alter the composition of the gut microbiota and lead to a slow growth rate. This study provides preliminary data support for understanding the biotoxicity of MPs to invertebrate zooplankton and is conducive to the further risk assessment of MP exposure.

## Introduction

Microplastics (MPs), as a new type of pollutant, have become one of the hotspots of studies by scientists in recent years. MPs are plastic wastes that broke down into smaller particles or plastic microspheres by processes such as ultraviolet radiation, waves, ocean current (Law et al., 2010), and microbial degradation (Cozar et al., 2014). They can enter the water environment through different pathways, like fishing plastic tools and marine litter transported from rivers, and through indirect pathways such as ocean currents and wind (Browne et al., 2007; Andrady, 2011).

Due to their inert nature and small size, MPs can present in the environment for a long time and be easily absorbed by surrounding organisms. They have been widely found in organisms such as black-horned algae, Asian clams, plankton (Su et al., 2016), corals (Hall et al., 2015), zooplankton (Setala et al., 2014), mussels, and oysters (Van Cauwenberghe and Janssen, 2014), as well as the fish in the English Channel (Lusher et al., 2013). MPs can be transferred between organisms at different trophic levels (Farrell and Nelson, 2013) and even translocated between various organs in the organism (Browne et al., 2008). Therefore, MPs have the potential to be transferred through the food chain to animals and humans, which will pose a serious threat to the safety of ecosystem and human health.

Upon digestion, MPs can accumulate in the worm’s intestine for a long time (Wright et al., 2013). Ziajahromi et al. (2018) evaluated the effects of four different sizes of polyethylene (PE) MPs on the growth and survival of Mucor larvae. Their results showed that MPs can affect the body length, survival, and reproduction of the animal. Lo and Chan (2018) used polystyrene (PS) microspheres to feed plankton larvae. Under the conditions of feeding the same number of algae, the growth rate of the larvae decreased significantly (Lo and Chan, 2018), which was consistent with this study. Previous studies have shown that MPs can affect the structure and abundance of intestinal flora. Xia et al. (2018) concluded that exposure of 1,000 μg⋅l^–1^ MPs will significantly reduce the abundance of Bacteroidetes and Proteobacteria in the zebrafish’s gut and increase the abundance of Firmicutes. Ju et al. (2019) found that PE can change the structure of gut microbiota and evidently reduce *Wolbachia* in the Proteobacteria in gut microbiota of soil collembola and increase the proportion of *Bradyrhizobiaceae*, *Ensifer*, and *Stenotrophomonas*.

Invertebrate zooplankton have an important role in the energy flow through trophic levels in different seawater systems. Brine shrimp (*Artemia parthenogenetica*) is a widely used model organism for toxicology research. It is a filter-feeding animal and can non-selectively filter a large amount of water per hour; therefore, it is more likely to be exposed to pollutants like MPs. Studies showed that a greater abundance of lipid droplets appeared in gut epithelia of brine shrimp after 24 h of PS exposure at a concentration of 10 particles/ml and intestinal epithelia were deformed and disorderedly arranged after 14 days of exposure (Wang et al., 2019a). Suman et al. (2020) reported that exposure to PS MPs in brine shrimp caused the upregulation of pyrimidodiazepine synthase and Crammer protein, indicating the increasing generation of ROS and accelerated apoptosis after exposure to PS MPs (Suman et al., 2020). Although there are some studies that reported the biotoxicity of MPs on brine shrimp, most of them are conducted with exposure of a high concentration of MPs within a short time, and there is lack of information on the gut microbiota change after long-term exposure with MPs.

Therefore, the purpose of this paper is to (1) investigate the effects of long-term MP exposure on the growth of brine shrimp with PE and PS MPs and (2) analyze the effects of long-term MP exposure on the structure and diversity of brine shrimp gut microbiota and discuss the relationship between MP exposure, growth inhibition, and the changes of microbial diversity. This is the first investigation on the intestinal microbial diversity change of brine shrimp after long-term MP exposure, and the evidence in this study will provide background information for MP toxicity studies and risk control strategies in future.

## Materials and Methods

### Materials

The Artemia cysts were purchased from Tianjin Haiyoujiayin Biotechnology Co., and kept frozen at −20°C until use. Artificial seawater was prepared by diluting the commercial sea salt (CNSIC Marine Biotechnology Co. Ltd., Jiaozhou, China) in purified water. During the experiment, a few grams of Artemia cysts were weighed and incubated in an incubator filled with artificial seawater. The artificial seawater was aerated for 2 h, of which the DO was controlled at 6.5 ± 0.5 mg/l and salinity at 32.3 ± 0.5‰. The pH was 8.5 ± 0.5, and the illumination was 2000 xl. PE and PS were purchased from Zhonglian Plastic Co. (Dongguan, China) with an average size of about 150 μm.

### Exposure Experiment

#### Artemia Growth Tests

To explore the effects of PE and PS MPs on the growth of brine shrimp, three treatments were set in this work, namely, CK (original seawater), PE (CK + 100 mg/l PE MPs, about 1.26 × 10^6^ items/m^3^), and PS (CK + 100 mg/l PS MPs, about 1.10 × 10^6^ items/m^3^). Each treatment had three replicates (*n* = 3). The content of 100 mg/l MPs was used in this study, which was also widely employed in other studies (Wang et al., 2019a,b). Artemia cysts (0.2 g) were hatched in a culture barrel for each treatment and were fed with 100 ml/day of Chlorella algal solution (∼1 × 10^5^ cells/ml). During the 45-day MP exposure, for each treatment, 10 brine shrimps were collected with a glass pipette every 3 days and observed under a microscope (BX53F, Olympus, Tokyo, Japan). The distribution of MPs in brine shrimp was observed under a stereomicroscope (SZX16, Olympus, Japan). The body length was measured, and the distribution of MPs in the gut was photographed by using cellSens Entry software (Olympus, Japan).

#### Microplastic Sample Analyses

Polyethylene and PS MPs used in this experiment were measured under a stereomicroscope (SZX16, Olympus, Japan). Brine shrimp guts were collected and digested for 3 h with 69% nitric acid at 70°C to isolate ingested MPs in the gut. FTIR analysis was conducted on a FTIR Spectrometer (IRAffinity-1, Shimadzu, Japan). Raman spectra were recorded by using a Raman spectrometer (Oceanhood, China) with a 785-nm laser. Laser excitation power was set to 200 mW, and integration time was set to 0.5 s. The surface morphology of MPs was observed through a FEI Quanta 650 FEG SEM (Thermo Fisher Scientific, Waltham, MA, United States) with an X-ray microanalysis device (EDS). For FTIR and EDS, both pristine and ingested MPs were measured.

#### Gut Microbiota’s 16S rRNA Gene Sequencing and Data Analyses

High-throughput sequencing of the 16S rRNA gene of gut microbiota was conducted on an Illumina MiSeq platform at Majorbio Genomics Institute (Shanghai, China). First, the gut of the brine shrimp was collected and transferred to a centrifuge tube. Then, DNA was extracted by using the E.Z.N.A. Soil Kit. PCR amplification of the v3–v4 variable region was conducted by using the universal primer 338F (5′-ACTCCTACGGGAGGCAGCAG-3′) and 806R (5′-GGACT ACHVGGGTWTCTAAT-3′). The amplification procedure was as follows: 95°C pre-denaturation for 3 min, followed by 29 cycles (95°C denaturation for 30 s, 55°C annealing for 30 s, 72°C extension for 30 s) and a final extension of 72°C for 10 min. The amplification system contained 20 μl with 4 μl 5 × FastPfu buffer, 2 μl 2.5 mM dNTPs, 0.8 μl primers (5 μM), 0.4 μl FastPfu polymerase, and 10 ng DNA template. The amplification products were recycled by using 2% agarose gels and then purified and eluted with Tris–HCl. Electrophoresis was performed using 2% agarose gels.

#### Statistical Analyses

The statistical analysis was performed using Excel and Origin software, and the FTIR spectra of MPs were analyzed using OMNIC software. The differences of Artemia’s body length and the growth rate of different treatments of MPs were analyzed by *t*-test, and the difference is significant at the 0.05 level.

Sequencing was performed on an Illumina’s Miseq PE300 platform (Shanghai Meiji Biomedical Technology Co., Ltd., Shanghai, China), and the raw data were uploaded to the NCBI database for comparison under the submission ID SUB9330660 and BioProject ID PRJNA716455. The quality of original sequences was controlled by Trimmomatic software and spliced by FLASH software. Based on the 97% similarity, the sequences were OTU clustered by UPARSE and chimeras were eliminated by UCHIME. The species classification of each sequence was annotated using the RDP classifier. We set a threshold of 70% and compared the sequences with the Silva database.

## Results

### Growth Inhibition and the Presence of Microplastics in the Gut

**FIGURE 1:**
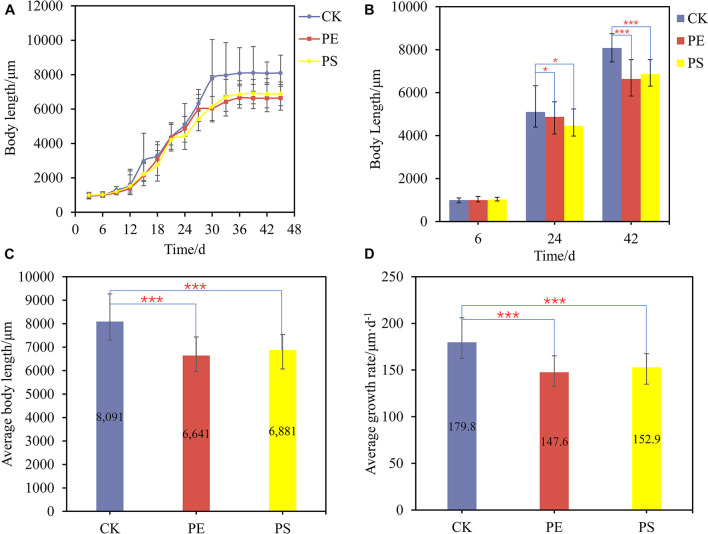
**(A)** Growth curves of brine shrimps treated with different MPs; **(B)** body length comparison between brine shrimps measured at 6, 24, and 42 days; comparison of average body length **(C)** and average growth rate **(D)** of brine shrimps treated with different MPs. Significance levels are indicated as *p < 0.05 and ***p < 0.005.

To explore the effect of MP exposure on the growth of brine shrimp, the body length of brine shrimp and the distribution of MPs in the gut were analyzed. The growth of brine shrimp went through a sluggish phase, an exponential growth phase, and a stable phase, namely, the first 10 days, from 10 to 36 days, and after 36 days (Figure 1A). In the sluggish period, there was no significant difference between the treatment group (PE and PS) and the CK group on the growth rate. However, there are remarkable differences between them in the exponential growth period. After 36 days, the growth of brine shrimp entered the stable phase and the body length reached the maximum. Figure 1B shows three typical points of different treatments which demonstrated the difference among the three growing stages.

The average body length of brine shrimps in CK, PE, and, PS was 8,091, 6,641, and 6,881 μm, respectively. The growth rate of brine shrimps in PE and PS was significantly different from that of CK (*p* < 0.05). Compared with CK, the growth rate of brine shrimps in PE and PS was 17.92 and 14.95% lower, respectively (Figure 1C). During the whole growth period from hatching to stabilization, the average growth rates of treatments CK, PE, and PS were 179.8, 147.6, and 152.9 μm/day, respectively (Figure 1D). The exposure of MPs decreased the average growth rate significantly, and the effect of the PS treatment is more serious.

**FIGURE 2:**
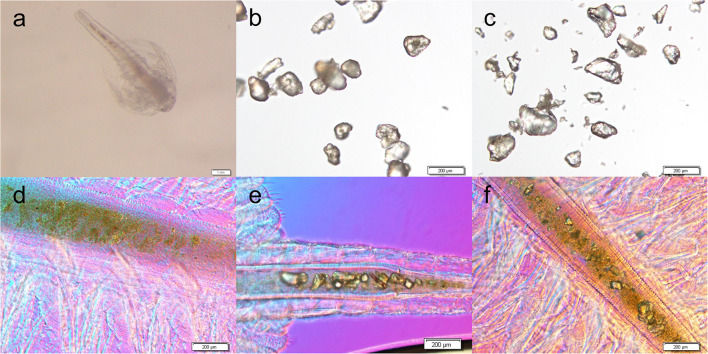
Microscopic photos of brine shrimp **(a)**, PE MPs **(b)**, and PS MPs **(c)**, and brine shrimp intestines of untreated CK **(d)**, treated with PE **(e)** and treated with PS **(f)**.

Figure 2 shows microscopic photos of brine shrimps and MPs. As is shown, MPs can be found in the intestine of brine shrimps after exposure with PE (Figure 2e) and PS (Figure 2f), while no MPs were found in CK (Figure 2d). In addition, there were no MPs distributed in other tissues. These data confirmed that MPs can be ingested by brine shrimp and distributed in the intestine.

### Characterization of Microplastics

**FIGURE 3:**
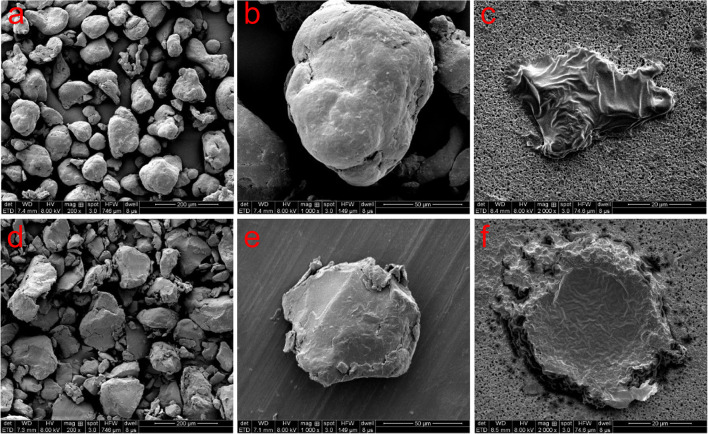
SEM images of pristine PE MPs **(a,b)**, extracted PE MPs **(c)**, pristine PS MPs **(d,e)**, and extracted PS MPs **(f)**.

**FIGURE 4:**
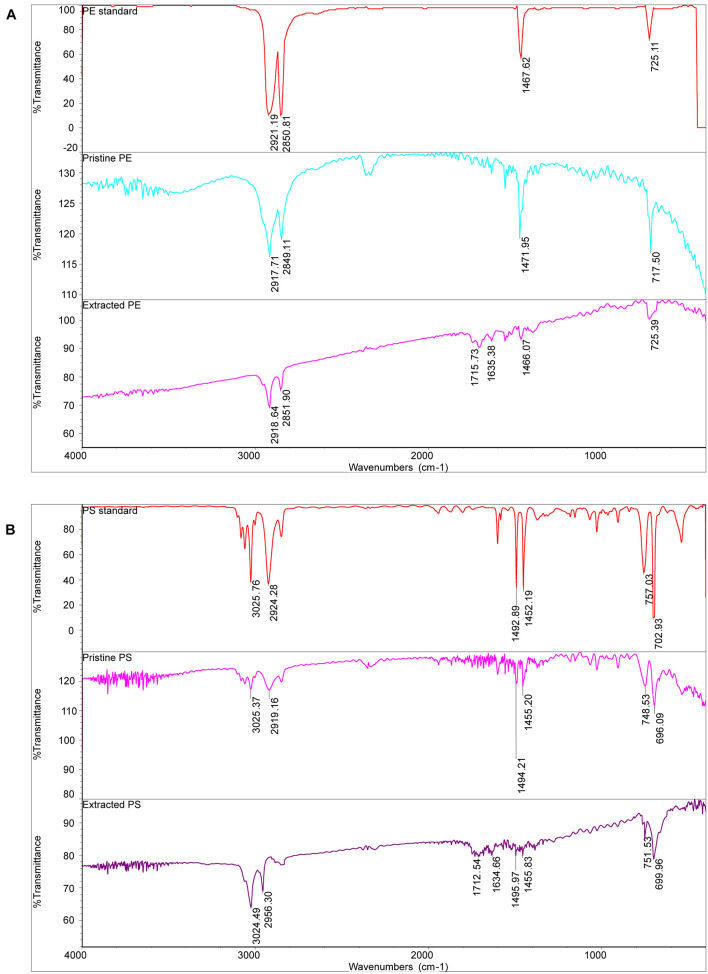
The FTIR spectra of PE MPs **(A)** and PS MPs **(B)**.

**FIGURE 5:**
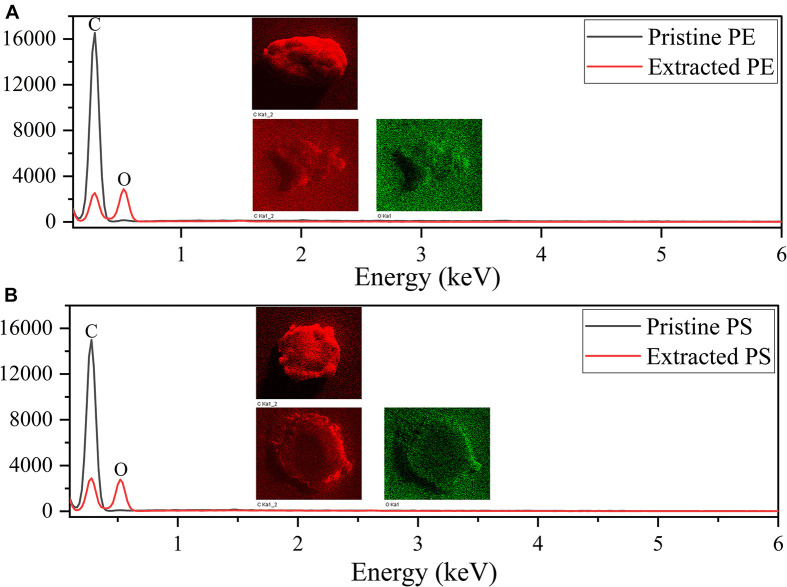
EDS spectra of PE MPs **(A)** and PS MPs **(B)**.

Figure 3 shows the SEM images of both pristine MPs and extracted MPs after ingestion. The company claimed that these MPs have an average size of about 150 μm; however, they are actually composed of small particles of uneven sizes (PE: from 40 to 220 μm, PS: from 30 to 300 μm). The surface of PE MPs was smoother than PS MPs. Figures 4A,B shows FTIR spectra of pristine and extracted MPs from shrimp gut in order to identify their compositions. The FTIR spectra of the pristine PE and PS MPs have identical patterns with PE and PS standards, respectively, confirming that there are no detectable additives in these chemicals. Raman analysis results are consistent with FTIR results (Supplementary Figure 1). The Raman spectrum for PE showed typical PE bands at 1,063.5, 1,129, 1,296, 1,440, 2,845, and 2,886 cm^–1^ (Fraser et al., 1995). The aromatic ring chain vibrations for PS at 1,000 and 1,600 cm^–1^ in Raman spectra were distinct (Kappler et al., 2016). The FTIR spectra of the extracted PE and PS MPs are consistent with those of the pristine MPs, demonstrating that the ingested particles are MPs. In the FTIR spectra of extracted PE and PS MPs, distinctive peaks located at around 1,715 and 1,635 cm^–1^ are found, representing the C=O group (Gieroba et al., 2020), which may generate by treatment by nitric acid during MP exaction. Figure 5 presents the energy-dispersive X-ray spectra of MPs. Elements of carbon and oxygen were abundant in extracted MPs while only carbon was abundant in pristine MPs, which was consistent with FTIR measurement.

### Dysbiosis of Gut Microbiota Caused by Exposure of Microplastics

#### Bacterial 16S rRNA Gene Sequencing

**FIGURE 6:**
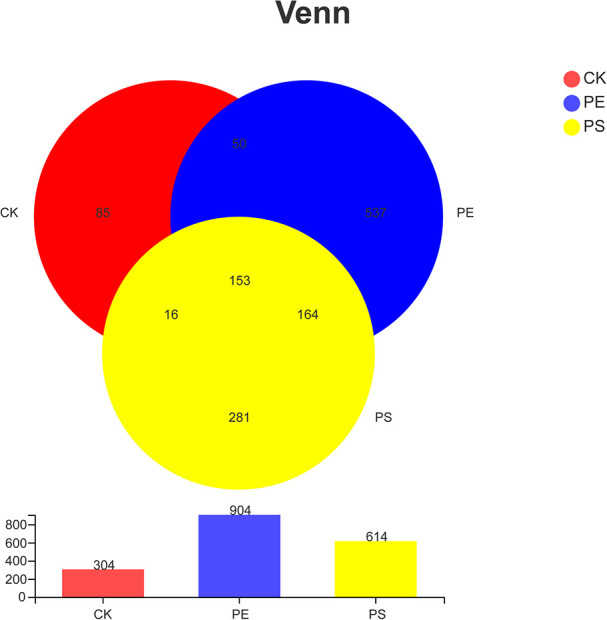
Venn diagram of the number of OTUs in different treatment groups. There are 304 OTUs in the control group, 904 OTUs in the PE group and 614 OTUs in PS group. The three samples share 153 OTUs, accounting for 50.33, 16.92, and 29.92%, respectively.

Table 1 indicates that the length of all the sequences obtained from the three samples (CK, PE, and PS) was between 205 and 408 bp. A total of 1,286 OTUs were obtained. To compare the diversity among the three groups, the Venn diagram was employed to show shared and unique communities (Figure 6). There were 153 common OTUs shared by the three groups. CK, PE, and, PS harbored 85, 537, and 281 unique OTUs, respectively.

**TABLE 1:** Sequencing data statistics for the different samples.

Sample	Seq_num	Base_num	Mean_length	Min_length	Max_length
CK	49480	20223553	408.7217664	259	446
PE	42965	17654323	410.9001047	205	480
PS	43889	18054846	411.3751965	250	441

#### Change of the Diversity of Intestinal Microbes

**FIGURE 7:**
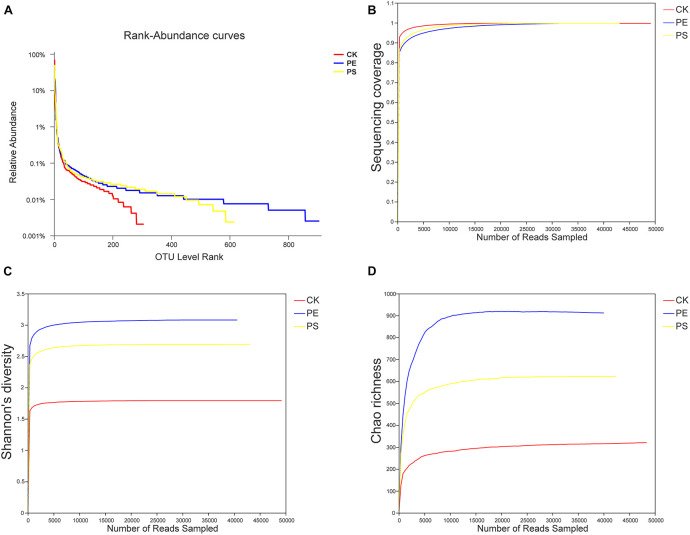
Changes of the diversity of intestinal microbes in different groups. **(A)** Rank-abundance curves, **(B)** sequencing coverage curves, **(C)** Shannon diversity curves, and **(D)** Chao richness curves.

The rank-abundance curve was drawn (Figure 7A) representing the species richness and evenness. The curve of CK was narrower and showed a sharp downward trend, indicating that the gut microbiota of brine shrimp had a lower diversity. However, PE and PS were more flat and wider, indicating a higher diversity. The Good’s coverage values ranged from 0.9363 to 0.9791 (Figure 7B). The dilution curve of all the three samples gradually flattens with the increase of the sequencing number, indicating that the sequencing depth was sufficient to cover all species from samples. The Shannon index curve tends to flatten with the increase of the number of sequencing lines, indicating that the amount of sequencing data is large enough to reflect the vast majority of taxa. As the amount of sequencing increased, the dilution curve was constantly flattening, indicating that the sequencing depth was sufficient to cover all taxa and reflect the diversity and richness of species and that the curves tended to saturation (Figure 7B). In comparing the Shannon diversity and Chao richness, the index of PE and PS increased remarkably compared with that of CK (Figures 7C,D). In addition, the increase of the Shannon and Chao indexes of PE was greater than that of PS.

#### The Composition of the Intestinal Microbial Structure of Brine Shrimp

With the relative abundance of species greater than 0.01 as the standard, the species composition was analyzed at the phylum and genus levels.

##### The structure on the *phylum* level

Four main phyla were detected in the community bar-plot analysis, namely, Proteobacteria, Actinobacteria, Firmicutes, and Bacteroidetes (Figure 9A), and the abundances of the four phyla in treatment CK, PE, and PS were (86.83, 34.92, 26.80), (8.68, 53.94, 63.63%), (1.47, 3.92, 4.07%), and (1.34, 4.07, 2.61%), respectively. Proteobacteria accounted for the largest proportion in treatment CK. However, Actinobacteria had the highest abundance in both treatment PE and PS. Besides, Firmicutes increased greater in treatment PE, and Bacteroidetes increased greater in treatment PS. The heat-map chart based on the phylum level showed that the four phyla in red account for the main proportion of gut microbiota, which was consistent with the results of the bar-plot analysis (Figure 9B). The flora of the PE and PS treatments’ cluster relationship was closer compared with treatment CK, which can be seen from the abundance of the flora.

##### The structure on the *genus* level

**FIGURE 8:**
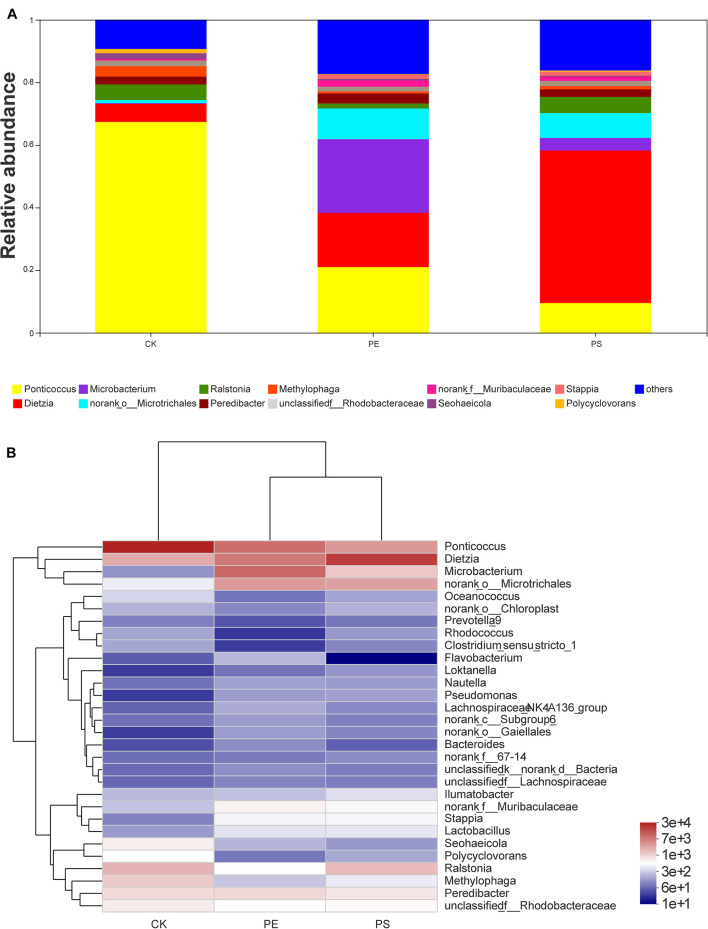
Relative abundance of intestinal microbiota at the genus level: **(A)** bar plot and **(B)** heat map chart.

The genera with abundance greater than 0.01 were *Ponticoccus*, *Dietzia*, *Microbacterium*, *Ralstonia*, *Peredibacter*, *Methylophaga*, *Seohaeicola*, *Stappia*, and *Polycyclovorans* (Figure 8A). The heat-map chart based on the genus level showed that the three treatments all had higher species richness (Figure 8B). Compared with treatment CK, MP treatments had lower abundance of *Ponticoccus* and higher abundance of *Dietzia*, *Microbacterium*, and *norank_o_Microtrichales*. *Ponticoccus* had the largest proportion in treatment CK as high as 67.36% and was relatively low in both MP treatments, which were lower than 25%. The top three genera with the highest abundance in treatment PE are *Microbacterium* (23.51%), *Ponticoccus* (20.98%), and *Dietzia* (17.37%), and its *Microbacterium* was the highest among the three treatments. The top three genera with the highest abundance in treatment PS are *Dietzia* (48.74%), *Ponticoccus* (9.49%), and *norank_o_Microtrichales* (7.89%), and *Dietzia* has the highest abundance among the three treatments. Both *Dietzia* and *Microbacterium* belong to *Actinomycetes*.

**FIGURE 9:**
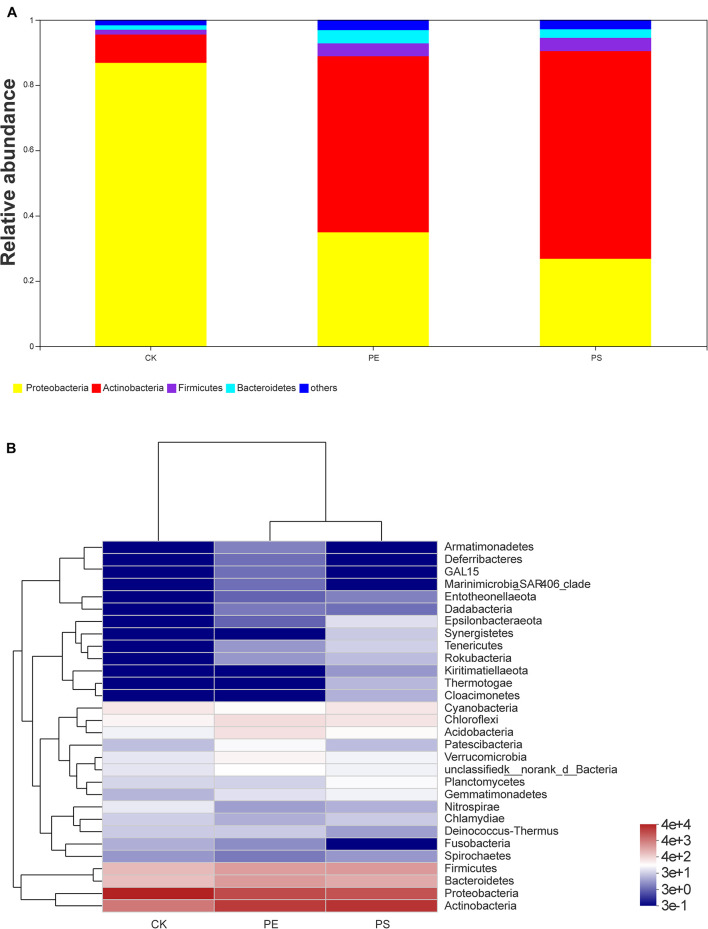
Relative abundance of intestinal microbiota at the phylum level: **(A)** bar plot and **(B)** heat map chart.

**FIGURE 10:**
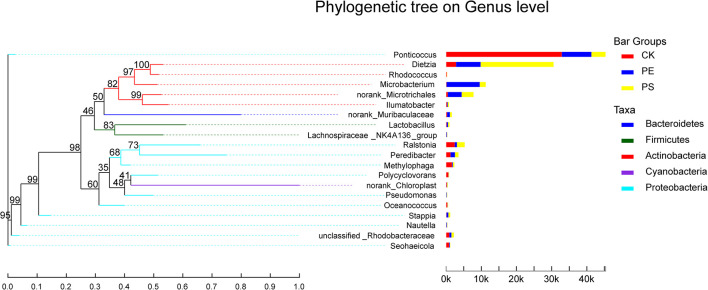
The phylogenetic tree of intestinal species at the genus level. It is based on the top 20 species in relative abundance. The left panel shows the phylogenetic tree, and the right panel denotes the species proportion relationship.

In the phylogenetic tree of intestinal species on the genus level (Figure 10), *Ponticoccus*, *Methylophaga*, and *Ralstonia* accounted for a relatively large proportion and their proportion in treatment CK was much larger than that in treatments PE and PS. These three bacterial genera’s evolutionary relationship was relatively close, and they all belong to the phylum Proteobacteria. *Dietzia*, *Microbacterium*, and *Microtrichales* had greater abundance in treatments PE and PS than treatment CK and had a close evolutionary relationship, and all belonged to the phylum Actinomycota.

## Discussion

### Impact of Microplastics on Brine Shrimp’s Growth

During the entire growth period, both PE and PS MPs significantly reduced the growth rate of brine shrimp and the average body length of adult brine shrimp. Previous studies had shown similar effects of MP exposure on other aquatic animals, including hippocampus and fish (Naidoo and Glassom, 2019; Sun et al., 2019). The remarkably altered growth rate and survival rate of test animals verified that the impact of MPs on the aquatic ecosystem was considerable. Our results illustrated that most of MPs were accumulated in the intestine and they can cause intestinal blocking which may reduce the feeding rate, finally leading to a lower growth rate of brine shrimp. Besides, the MPs that remained in the gut may damage the epithelial cells of the digestive tract. Previous reports have evidenced that MPs can accumulate in various aquatic organisms, including fish and zooplankton (Lusher et al., 2015; Kokalj et al., 2018). Mattsson et al. (2015) found that intake of MPs can decrease the sport and feeding activities of black crucian carp. The ingestion of MPs was also found reducing the feeding rate of *Daphnia magna* and copepod plankton (Barnes et al., 2009; Cole et al., 2013; Rist et al., 2017), and decreasing the number of microvilli in epithelial cells of *A. parthenogenetica* (Wang et al., 2019b). MPs are also reported causing liver inflammation and lipid accumulation in zebrafish, affecting lipid and energy metabolism (Lu et al., 2016). In comparison to that of PS MPs, PE MPs had a greater inhibition effect on the growth rate of brine shrimp. After entering the stable growth stage, the average growth rate of brine shrimps in the PE group was smaller than that of brine shrimps in the PS group. This phenomenon demonstrated that PS and PE had different toxicities toward brine shrimp. Therefore, the different physiochemical properties and environmental behavior MPs may be the reason why they have different toxic effects on aquatic animals. As a whole, the intake of MPs can bring physical blocking and biochemical toxicity to brine shrimps and may lead to damage of intestinal epithelial cells, which caused intestinal inflammation and altered the material and energy metabolism in the brine shrimp body, resulting in the inhibition of brine shrimp’s growth.

### Impact of Microplastics on Brine Shrimp’s Gut Microbiota

The type of host feeding is a key factor affecting the diversity of the gut microbiota (Voreades et al., 2014). PE and PS MPs increased the abundance of the gut microbiota of brine shrimp. It is shown in Figures 7, 9 that the presence of PE and PS shifted the structure of brine shrimp gut microbiota and increased the microbial diversity. Among the four main microbial phyla detected in the gut of brine shrimp, the proportion of Proteobacteria under MP exposure was reduced, and the proportions of Actinomycetes and Bacteroides were significantly increased. The sharp increase of Bacteroides led to an imbalance between microbial populations, which may affect the material metabolism process of brine shrimp and caused its slower growth. This result is consistent with a previous report on zebrafish. Wan et al. found that PS particles can significantly affect the gut microbiota in both larval zebrafish and adult zebrafish. MP exposure changes the ratio of Bacteroides to Firmicutes, affecting energy metabolism, glucose metabolism, and lipid metabolism, interfering the growth of zebrafish (Wan et al., 2019). The presence of balanced gut microbiota plays an important role in the animal growth and health. The number of total bacteria, Firmicutes, Proteobacteria, and Actinobacteria can affect the growth and disease resistance of fishes (Teame et al., 2020). This study confirmed that changes in the gut microbiota caused by MPs will affect the growth and development of aquatic organisms.

According to the analysis at the *genus* level, the proportions of *Ponticoccus*, *Seohaeicola*, and *Stappia* in phylum Proteobacteria decreased, and the proportions of *Methylophaga* and *Polycyclovorans* in phyla Proteobacteria, *Microbacterium*, and *Dietzia* in phylum Actinomycota increased (Figure 8). *Dietzella* has been reported to be related to human diseases (Bemer-Melchior et al., 1999), and infection of *Dietzella* can lead to symptoms of pneumothorax and septic shock. There is also evidence that infection of pine wood nematodes by *Actinomycetes* can impair their movement ability and remarkably increase the mortality (Liu et al., 2019). Therefore, it was reasonable to speculate that the increase of *Dietzella* may impair the intestinal health of brine shrimp. *Methylophaga* and *Polycyclovorans* have been reported to play roles on the degradation of hexose and pentose oligomers. The decrease of *Methylophaga* and *Polycyclovorans* may limit the utilization of carbon sources for brine shrimp and therefore slow down its growth rate (Wang et al., 2020). In addition, studies have shown that the uncontrolled augmentation of Proteobacteria can facilitate inflammation or invasion by exogenous pathogens (Shin et al., 2015). As to genus *Stappia*, bacteria of this genus have been isolated from the various habitats (Kim et al., 2006; Kumar et al., 2011; Kampfer et al., 2013), and they are found to participate in various processes, such as viral defense, secondary metabolite production, polyamine metabolism, polypeptide uptake membrane transport, and putative energy neutral pressure-dependent CO_2_ fixation (Vick et al., 2020). There were few reports on the function of genera *Ponticoccus*, *Seohaeicola*, and *Stappia* in the organisms’ gut; their potential roles merit further investigation. In a word, MP exposure can alter the structure of gut microbiota, break the balance between the populations, and even create an environment conducive to the growth of pathogens. These changes can further disturb the metabolism of material and energy and are supposed to be the main reasons for affecting the growth of brine shrimps.

**FIGURE 11:**
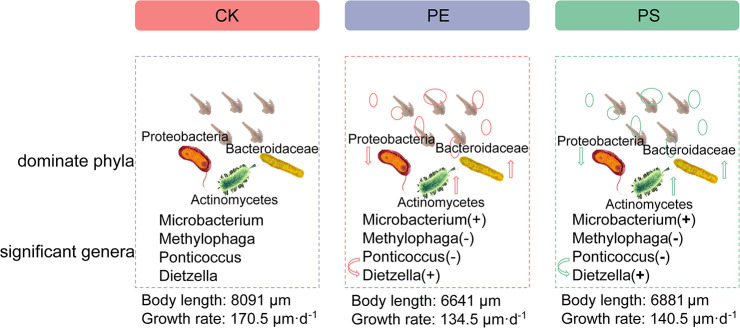
Schematic of the effect of MP exposure on composition of intestinal microbiota (dominant phyla, significant genera) and growth of brine shrimp. “+” means high, and “−” means low.

Our results preliminarily demonstrated that there was a link between the composition changes of the shrimp’s gut microbiota and the slow growth rate. Figure 11 showed the schematic of the effect of MP exposure on the growth and the intestinal microbiota composition of brine shrimp. The disorder degree of gut microbiota was consistent with growth rate inhibition: PE MP exposure induced a more severe disorder of gut microbiota and led to a greater inhibition on the growth rate. The microbial composition at the phylum level for each treatment was changed with the similar trend. However, there exist many factors that impact the growth rate of brine shrimps. The roles of gut microbiota involved in this impact can be further revealed by various other methods, such as meta-transcriptomic and meta-genomic analyses. In addition, although this work and the increasing body of literature on MPs have demonstrated the existence of the relationship between altered growth rate and gut microbiota, the detail mechanisms need to be further explored.

## Conclusion

We studied the influence of long-term exposure of MPs (PE and PS) on the growth of brine shrimp (*A. parthenogenetica*), and a preliminary investigation on the gut microbiota was conducted to explore the reason for inhibition of growth rate by exposure of MPs. Our current work demonstrated that the long-term exposure of brine shrimp to MPs significantly inhibited the growth rate of brine shrimp. Different MPs have different impacts on the growth of brine shrimp. High-throughput sequencing results revealed that MP exposure significantly changed the diversity of gut microbiota. The disorder degree of gut microbiota was consistent with growth rate inhibition: PE MP exposure induced a more severe disorder of gut microbiota and led to a greater inhibition on the growth rate. The microbial composition at the phylum level for each treatment was changed with the similar trend. The relative abundance of *Ponticoccus*, *Seohaeicola*, and *Stappia* in phylum Proteobacteria was remarkably decreased, and the relative abundance of *Methylophaga* and *Polycyclovorans* in phyla Proteobacteria, *Microbacterium*, and *Dietzia* in phylum Actinomycota was evidently increased. The alteration of the microbial community structure may disturb energy harvesting and storage and have an adverse impact on the intestinal and body health of brine shrimp, thereby inhibiting its growth. These findings shed new insights into the toxic effects of MPs on brine shrimps and provide an experimental basis for the risk assessment and control of marine plastic debris.

## Data Availability Statement

The datasets presented in this study can be found in online repositories. The names of the repository/repositories and accession number(s) can be found in the article/Supplementary Material.

## Author Contributions

HL, HC, JW, JL, SL, PZ, XL, and ZW developed the concept for this study. HL, HC, JW, and XL designed the experiments. HL and HC performed the microbiological experiments and drafted the manuscript. HL, HC, JL, SL, and XL contributed the reagents, materials, and analysis tools. All authors interpreted the results, edited and reviewed the final version of the manuscript, and approved it before submission.

## Conflict of Interest

The authors declare that the research was conducted in the absence of any commercial or financial relationships that could be construed as a potential conflict of interest.

## Publisher’s Note

All claims expressed in this article are solely those of the authors and do not necessarily represent those of their affiliated organizations, or those of the publisher, the editors and the reviewers. Any product that may be evaluated in this article, or claim that may be made by its manufacturer, is not guaranteed or endorsed by the publisher.

## References

[B1] Andrady A. L. (2011). Microplastics in the marine environment. *Mar. Pollut. Bull.* 62 1596–1605. 10.1016/j.marpolbul.2011.05.030 21742351

[B2] Barnes D. K. A. Galgani F. Thompson R. C. Barlaz M. (2009). Accumulation and fragmentation of plastic debris in global environments. *Philos. Trans. R. Soc. B Biol. Sci.* 364 1985–1998. 10.1098/rstb.2008.0205 19528051 PMC2873009

[B3] Bemer-Melchior P. Haloun A. Riegel P. Drugeon H. B. (1999). Bacteremia due to *Dietzia maris* in an immunocompromised patient. *Clin. Infect. Dis.* 29 1338–1340. 10.1086/313490 10524995

[B4] Browne M. A. Dissanayake A. Galloway T. S. Lowe D. M. Thompson R. C. (2008). Ingested microscopic plastic translocates to the circulatory system of the mussel, *Mytilus edulis* (L.). *Environ. Sci. Technol.* 42 5026–5031. 18678044 10.1021/es800249a

[B5] Browne M. A. Galloway T. Thompson R. (2007). Microplastic—an emerging contaminant of potential concern? *Intergtr. Environ. Assess. Manag.* 3 559–561. 10.1002/ieam.5630030412 18046805

[B6] Cole M. Lindeque P. Fileman E. Halsband C. Goodhead R. Moger J. (2013). Microplastic Ingestion by Zooplankton. *Environ. Sci. Technol.* 47 6646–6655. 10.1021/es400663f 23692270

[B7] Cozar A. Echevarria F. Gonzalez-Gordillo J. I. Irigoien X. Ubeda B. Hernandez-Leon S. (2014). Plastic debris in the open ocean. *Proc. Natl. Acad. Sci. U.S.A.* 111 10239–10244. 10.1073/pnas.1314705111 24982135 PMC4104848

[B8] Farrell P. Nelson K. (2013). Trophic level transfer of microplastic: *Mytilus edulis* (L.) to Carcinus maenas (L.). *Environ. Pollut.* 177 1–3. 10.1016/j.envpol.2013.01.046 23434827

[B9] Fraser G. V. Hendr P. J. Cudby M. E. A. Willi H. A. (1995). Laser-raman spectrum of polyethylene under high pressure. *J. Chem. Soc. Chem. Commun.* 1, 16–17.

[B10] Gieroba B. Krysa M. Wojtowicz K. Wiater A. Pleszczynska M. Tomczyk M. (2020). The FT-IR and raman spectroscopies as tools for biofilm characterization created by cariogenic Streptococci. *Int. J. Mol. Sci.* 21:3811. 10.3390/ijms21113811 32471277 PMC7313032

[B11] Hall N. M. Berry K. L. E. Rintoul L. Hoogenboom M. O. (2015). Microplastic ingestion by scleractinian corals. *Mar. Biol.* 162 725–732. 10.1007/s00227-015-2619-7

[B12] Ju H. Zhu D. Qiao M. (2019). Effects of polyethylene microplastics on the gut microbial community, reproduction and avoidance behaviors of the soil springtail, Folsomia candida. *Environ. Pollut.* 247 890–897. 10.1016/j.envpol.2019.01.097 30735918

[B13] Kampfer P. Arun A. B. Frischmann A. Busse H. J. Young C. C. Rekha P. D. (2013). *Stappia taiwanensis* sp. nov., isolated from a coastal thermal spring. *Int. J. Sys. Evol. Microbiol.* 63(Pt 4), 1350–1354. 10.1099/ijs.0.044966-0 22798655

[B14] Kappler A. Fischer D. Oberbeckmann S. Schernewski G. Labrenz M. Eichhorn K. J. (2016). Analysis of environmental microplastics by vibrational microspectroscopy: FTIR, Raman or both? *Anal. Bioanal. Chem.* 408 8377–8391. 10.1007/s00216-016-9956-3 27722940

[B15] Kim B. C. Park J. R. Bae J. W. Rhee S. K. Kim K. H. Oh J. W. (2006). *Stappia marina* sp. nov., a marine bacterium isolated from the Yellow Sea. *Int. J. Syst. Evol. Microbiol.* 56(Pt 1), 75–79. 10.1099/ijs.0.63735-0 16403869

[B16] Kokalj A. J. Kunej U. Skalar T. (2018). Screening study of four environmentally relevant microplastic pollutants: uptake and effects on *Daphnia magna* and *Artemia franciscana*. *Chemosphere* 208 522–529. 10.1016/j.chemosphere.2018.05.172 29890490

[B17] Kumar A. P. Srinivas T. N. R. Madhu S. Shivaji S. (2011). *Lutibaculum baratangense* gen. nov., sp. nov., a novel proteobacterium isolated from a mud volcano, Andamans, India. *Int. J. Syst. Evol. Microbiol.* 61:1763. 10.1099/ijs.0.027094-0 21148673

[B18] Law K. L. Morét-Ferguson S. Maximenko N. A. Proskurowski G. Peacock E. E. Hafner J. (2010). Plastic accumulation in the North Atlantic Subtropical Gyre. *Science* 329 1185–1188. 20724586 10.1126/science.1192321

[B19] Liu M. J. Hwang B. S. Jin C. Z. Li W. J. Park D. J. Seo S. T. (2019). Screening, isolation and evaluation of a nematicidal compound from actinomycetes against the pine wood nematode. *Bursaphelenchus xylophilus*. *Pest Manag. Sci.* 75 1585–1593. 10.1002/ps.5272 30461185

[B20] Lo H. K. A. Chan K. Y. K. (2018). Negative effects of microplastic exposure on growth and development of *Crepidula onyx*. *Environ. Pollut.* 233 588–595. 10.1016/j.envpol.2017.10.095 29107898

[B21] Lu Y. F. Zhang Y. Deng Y. F. Jiang W. Zhao Y. P. Geng J. J. (2016). Response to Comment on “Uptake and Accumulation of Polystyrene Microplastics in Zebrafish (*Danio rerio*) and Toxic Effects in Liver”. *Environ. Sci. Technol.* 50 12523–12524. 10.1021/acs.est.6b04379 27808508

[B22] Lusher A. L. McHugh M. Thompson R. C. (2013). Occurrence of microplastics in the gastrointestinal tract of pelagic and demersal fish from the English Channel. *Mar. Pollut. Bull.* 67 94–99. 10.1016/j.marpolbul.2012.11.028 23273934

[B23] Lusher A. L. Tirelli V. O’Connor I. Officer R. (2015). Microplastics in Arctic polar waters: the first reported values of particles in surface and sub-surface samples. *Sci. Rep.* 5:14947. 10.1038/srep14947 PMC4597356 26446348

[B24] Mattsson K. Ekvall M. T. Hansson L. A. Linse S. Malmendal A. Cedervall T. (2015). Altered behavior, physiology, and metabolism in fish exposed to polystyrene nanoparticles. *Environ. Sci. Technol.* 49 553–561. 10.1021/es5053655 25380515

[B25] Naidoo T. Glassom D. (2019). Decreased growth and survival in small juvenile fish, after chronic exposure to environmentally relevant concentrations of microplastic. *Mar. Pollut. Bull.* 145 254–259. 10.1016/j.marpolbul.2019.02.037 31590784

[B26] Rist S. Baun A. Hartmann N. B. (2017). Ingestion of micro- and nanoplastics in *Daphnia magna* - Quantification of body burdens and assessment of feeding rates and reproduction. *Environ. Poll.* 228 398–407. 10.1016/j.envpol.2017.05.048 28554029

[B27] Setala O. Fleming-Lehtinen V. Lehtiniemi M. (2014). Ingestion and transfer of microplastics in the planktonic food web. *Environ. Pollut.* 185 77–83. 10.1016/j.envpol.2013.10.013 24220023

[B28] Shin N. R. Whon T. W. Bae J. W. (2015). *Proteobacteria*: microbial signature of dysbiosis in gut microbiota. *Trends Biotechnol.* 33 496–503. 10.1016/j.tibtech.2015.06.011 26210164

[B29] Su L. Xue Y. G. Li L. Y. Yang D. Q. Kolandhasamy P. Li D. J. (2016). Microplastics in Taihu Lake. China. *Environ. Pollut.* 216 711–719. 10.1016/j.envpol.2016.06.036 27381875

[B30] Suman T. Y. Jia P. P. Li W. G. Junaid M. Xin G. Y. Wang Y. (2020). Acute and chronic effects of polystyrene microplastics on brine shrimp: first evidence highlighting the molecular mechanism through transcriptome analysis. *J. Hazard. Mater.* 400:123220. 10.1016/j.jhazmat.2020.123220 32590134

[B31] Sun J. H. Xia S. D. Ning Y. Pan X. Qu J. H. Xu Y. J. (2019). Effects of microplastics and attached heavy metals on growth, immunity, and heavy metal accumulation in the yellow seahorse, Hippocampus kuda Bleeker. *Mar. Polluti. Bull.* 149:110510. 10.1016/j.marpolbul.2019.110510 31450030

[B32] Teame T. Wu X. Hao Q. Ding Q. Liu H. Ran C. (2020). Dietary SWF§enhanced growth performance and disease resistance in hybrid sturgeon (*Acipenser baerii* x *Acipenser schrenckii*) mediated by the gut microbiota. *Aquacul. Rep.* 17:100346. 10.1016/j.aqrep.2020.100346

[B33] Van Cauwenberghe L. Janssen C. R. (2014). Microplastics in bivalves cultured for human consumption. *Environ. Pollut.* 193 65–70. 10.1016/j.envpol.2014.06.010 25005888

[B34] Vick S. H. W. Greenfield P. Willows R. D. Tetu S. G. Midgley D. J. Paulsen I. T. (2020). Subsurface *Stappia*: success through defence, specialisation and putative pressure-dependent carbon fixation. *Microb. Ecol.* 80 34–46. 10.1007/s00248-019-01471-y 31828390

[B35] Voreades N. Kozil A. Weir T. L. (2014). Diet and the development of the human intestinal microbiome. *Front. Microbiol.* 5:494. 10.3389/fmicb.2014.00494 25295033 PMC4170138

[B36] Wan Z. Wang C. Zhou J. Shen M. Wang X. Fu Z. (2019). Effects of polystyrene microplastics on the composition of the microbiome and metabolism in larval zebrafish. *Chemosphere* 217 646–658. 10.1016/j.chemosphere.2018.11.070 30448747

[B37] Wang S. Wang L. Fan X. Yu C. Feng L. Yi L. (2020). An insight into diversity and functionalities of gut microbiota in insects. *Curr. Microbiol.* 77 1976–1986. 10.1007/s00284-020-02084-2 32535651

[B38] Wang Y. Mao Z. Zhang M. Ding G. Sun J. Du M. (2019a). The uptake and elimination of polystyrene microplastics by the brine shrimp, *Artemia parthenogenetica*, and its impact on its feeding behavior and intestinal histology. *Chemosphere* 234 123–131. 10.1016/j.chemosphere.2019.05.267 31207418

[B39] Wang Y. Zhang D. Zhang M. Mu J. Ding G. Mao Z. (2019b). Effects of ingested polystyrene microplastics on brine shrimp, *Artemia parthenogenetica*. *Environ. Pollut.* 244 715–722. 10.1016/j.envpol.2018.10.024 30384077

[B40] Wright S. L. Rowe D. Thompson R. C. Galloway T. S. (2013). Microplastic ingestion decreases energy reserves in marine worms. *Curr. Biol.* 23 R1031–R1033. 10.1016/j.cub.2013.10.068 24309274

[B41] Xia J. Lu L. Jin C. Wang S. Zhou J. Ni Y. (2018). Effects of short term lead exposure on gut microbiota and hepatic metabolism in adult zebrafish. *Comp. Biochem. Physiol.* 209 1–8. 10.1016/j.cbpc.2018.03.007 29574035

[B42] Ziajahromi S. Kumar A. Neale P. A. Leusch F. D. L. (2018). Environmentally relevant concentrations of polyethylene microplastics negatively impact the survival, growth and emergence of sediment-dwelling invertebrates. *Environ. Pollut.* 236 425–431. 10.1016/j.envpol.2018.01.094 29414367

