# Supplementary material for: Influence of Microplastics on the Growth and the Intestinal Microbiota Composition of Brine Shrimp
Source: Front Microbiol. 2021 Sep 29;12:717272. doi: 10.3389/fmicb.2021.717272 (PMC8511709; doi:10.3389/fmicb.2021.717272)
Supplement: Supplementary file 1 [file Data_Sheet_1.docx]

Supplementary Information

Influence of microplastics on the growth and the intestinal microbiota composition of brine shrimp

**Hongyu Li^1^, Hongwei Chen^1^, Jiao Wang^1^, Jiayao Li^1^, Sitong Liu^1^, Jianpo Tu^2^, Yanzhen Chen^2^, Yanping Zong^2^, Pingping Zhang^3^, Zhiyun Wang^1*^ and Xianhua Liu^1*^**

^1^School of Environmental Science and Engineering, Tianjin University, Tianjin, 300354, PR China

^2^Tianjin Marine Environment Monitoring Center Station of State Oceanic Administration, Tianjin, 300457, PR China

^3^College of Food Science and Engineering, Tianjin Agricultural University, Tianjin, 300384, PR China

*** Correspondence:**Zhiyun Wang, Xianhua Liu
zhiyun_wang@tju.edu.cn, [lxh@tju.edu.cn](mailto:lxh@tju.edu.cn)

## Supplementary Figures


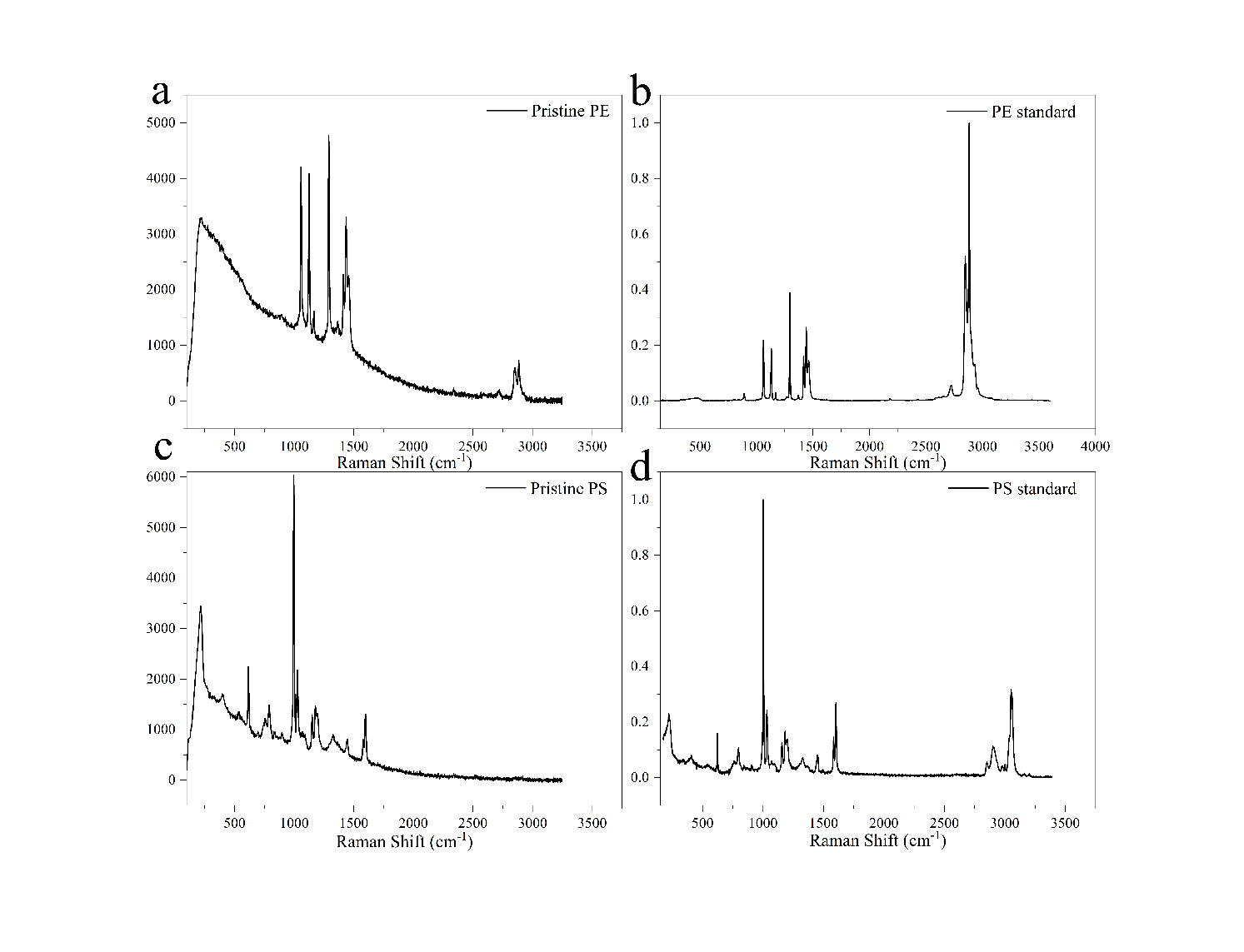


**Figure S1.** Raman spectra of pristine PE MPs (a), PE standard (b), pristine PS MPs (c) and PS standard (d).
